# A meta-model for computer executable dynamic clinical safety checklists

**DOI:** 10.1186/s12911-017-0551-0

**Published:** 2017-12-12

**Authors:** Shan Nan, Pieter Van Gorp, Xudong Lu, Uzay Kaymak, Hendrikus Korsten, Richard Vdovjak, Huilong Duan

**Affiliations:** 1 School of Biomedical Engineering and Instrumental Science, Zhejiang University, Hangzhou, China; 2 School of Industrial Engineering, Eindhoven University of Technology, Eindhoven, The Netherlands; 3 Anesthesiology and Intensive Care, Catharina Ziekenhuis in Eindhoven, Eindhoven, The Netherlands; 4 Philips Research Europe, Eindhoven, The Netherlands

**Keywords:** Checklist, Metamodel, BPMN, GLIF, GASTON, Drools

## Abstract

**Background:**

Safety checklist is a type of cognitive tool enforcing short term memory of medical workers with the purpose of reducing medical errors caused by overlook and ignorance. To facilitate the daily use of safety checklists, computerized systems embedded in the clinical workflow and adapted to patient-context are increasingly developed. However, the current hard-coded approach of implementing checklists in these systems increase the cognitive efforts of clinical experts and coding efforts for informaticists. This is due to the lack of a formal representation format that is both understandable by clinical experts and executable by computer programs.

**Methods:**

We developed a dynamic checklist meta-model with a three-step approach. Dynamic checklist modeling requirements were extracted by performing a domain analysis. Then, existing modeling approaches and tools were investigated with the purpose of reusing these languages. Finally, the meta-model was developed by eliciting domain concepts and their hierarchies. The feasibility of using the meta-model was validated by two case studies. The meta-model was mapped to specific modeling languages according to the requirements of hospitals.

**Results:**

Using the proposed meta-model, a comprehensive coronary artery bypass graft peri-operative checklist set and a percutaneous coronary intervention peri-operative checklist set have been developed in a Dutch hospital and a Chinese hospital, respectively. The result shows that it is feasible to use the meta-model to facilitate the modeling and execution of dynamic checklists.

**Conclusions:**

We proposed a novel meta-model for the dynamic checklist with the purpose of facilitating creating dynamic checklists. The meta-model is a framework of reusing existing modeling languages and tools to model dynamic checklists. The feasibility of using the meta-model is validated by implementing a use case in the system.

## Background

In recent years, safety checklists have been developed in response to the growing number of preventable medical errors [1]. These checklists seek to improve adherence to best practices for error-prone activities in healthcare processes by providing visual or verbal guiding [2]. Although various well-established studies [3, 4] have proven that they can improve the quality of care significantly in experimental environments, they have not yet effectively changed clinical practices [5]. One major reason is the additional workload imposed on the care-givers due to the poor integration into their daily practice and routines [6, 7].

To foster the frequent and effective use of these checklists in daily practice, clinical decision support enhanced checklists are increasingly being developed and implemented [8–10]. For example, some checklists can provide active reminders at the right time in the clinical workflow [11], automatically filter of checklist items [10–12] and provide pertinent medical literature and patient data [8, 9, 11]. A computerized checklist enhanced by clinical decision support features is characterized as a smart checklist or dynamic checklist [11, 12]. These dynamic checklists can improve the adherence significantly while reducing care-givers’ workload compared to the “static” checklists [9].

Currently, most dynamic checklists are hard-coded and tightly coupled with specific information systems (typically Electronic Medical Record Systems). Though hard-code approach is rather straightforward, we argue that it has several insufficiencies. Firstly, clinical experts and users do not understand the encoding approach nor the encoded knowledge if it is in the form of software source code. However, clinical experts’ involvement is critical for the success of checklist. Unnecessary communication cost and potential communication errors are introduced into this approach. Secondly, the definition of these dynamic checklists is tightly coupled with their execution. It makes the definition (i.e., model) difficult to be extended, maintained and reused. Additionally, guideline-based clinical decision support and workflow management research in recent years already provide solutions in their problem domain for similar problems. The hard-coded approach can not take advantage of these methodologies and knowledge.

This is undesirable, especially since encoding clinical knowledge in executable and shareable formats has been studied and practiced for decades [13, 14]. For example, clinical guideline modeling languages and workflow modeling languages have been developed for and applied to encoding clinical guidelines and clinical pathways. Knowledge acquisition tools and execution engines are also developed to facilitate the use of these modeling languages. The languages together with the tools enable clinical users encoding guidelines and pathways and sharing the encoded knowledge among hospitals.

It would be ideal if dynamic checklists can take the advantage of such modeling languages, tools, and encoded knowledge. Prior research considering checklists as a modeling construct in clinical workflow modeling has already contributed partial efforts towards this direction. Fäber et al. [15] have demonstrated the meaningful use of parallel tasks for the modeling of checklist-supported workflows. However, it was not yet investigated how to deal for example with dynamicity in checklist forms, i.e., representing clinical algorithms. Heß et al. [16] have proposed a clinical pathway domain-specific modeling language, which considers checklist as a supportive concept in medical processes. In their research, a checklist is further decomposed into patient specific checklist elements. However, it was not revealed how the modelers could define in which context a patient specific element should be used. Further study is needed to fully answer the research question by developing a conceptual model describing the complete structural and functional requirements of dynamic checklists.

Because dynamic checklists have the function of managing healthcare processes, personalizing checklist items and extracting data out of the patient record, it is likely that one single existing modeling language can not fully support representing the dynamic checklists. Instead, multiple languages may be used comprehensively. In this case, it becomes a challenge how we can integrate multiple modeling languages for the purpose of representing dynamic checklists.

In this paper, we answer these research questions by contributing a meta-model formalizing the modeling requirements and emphasizing the interoperability between modeling languages. With this meta-model, the clinical users choose and use their familiar modeling languages and editors. The execution of the model can also take the advantage by using the implemented execution engines. As a result, the notion of the dynamic checklist can be better adopted and widely used. Furthermore, we have the ambition to enable researchers and vendors sharing their dynamic checklist models worldwide so that they can reuse, validate and compare models developed by others. In such a way, the implementation of dynamic checklists can be accelerated.

## Methods

In this section, we demonstrate how the dynamic checklist meta-model was developed and validated.

### Develop the meta-model

Referring to [17], we developed a three-step approach to derive our dynamic checklist meta-model. Firstly, a problem domain analysis was performed to clarify what makes dynamic checklists dynamic. Then, we investigated existing modeling approaches with the purpose of taking advantage of mature tools. Finally, we developed the meta-model by eliciting dynamic checklist related concepts, determining the class hierarchies and properties.

#### Problem domain analysis: scope of dynamic checklists

Efforts on developing and implementing dynamic checklists have been increasingly reported in recent years. Academic Medical Center (AMC) has rolled out their SURgical Patient Safety System (SURPASS) Digital since 2011 [18], which is the computerized version of their well-known SURPASS checklist [4]. SURPASS Digital aims to better streamline the process of using SURPASS checklists for each patient and ease the use for care-givers. To streamline the process, SURPASS Digital uses a web-based user interface integrated with their EMR. Once a patient is selected in the EMR, a care-giver can overview the status and results of all the checklists or pick up a checklist to work with. The integration is supported in the back-end by an information integration platform specific to AMC to retrieve patient registries and other information from the EMR. In the front-end, programs like JavaScript are used to improve the interaction and validate the checking logic.

While SURPASS Digital mainly focuses on streamlining the process, other research on encouraging using checklists in specific scenarios are increasingly reported. For example, a computerized discharge checklist is reported by Stanford University Medical Center [10]. In their system, the paper discharge checklist is implemented as an Electronic Medical Record System (EMR) specific keyword. Once a user types the keyword “.dcchecklist”, the system automatically inserts a predefined checklist template into the patient’s EMR chart. In addition to this, many researchers now are focusing on making a checklist adaptive to specific patients. Two dynamic checklists for the intensive care unit (ICU) are reported by Mayo Clinic [9] and Lucile Packard Children’s Hospital at Stanford [8] respectively. Both of them are integrated with EMR, and some of their content is derived from or calculated by clinical rules. For example, for a patient who has been placed with a central line for multiple days, there will be a red item added in the checklist warning the intensivist to evaluate the necessity of keeping these lines placed.

Our research team has also reported the efforts on developing a comprehensive dynamic checklist support system named Tracebook [11]. It covers both streaming the process and personalizing the checklists. Tracebook is designed to be a checklist execution platform for dynamic checklists which are process-oriented and patient context-aware. In Tracebook, each checklist is associated with a clinical activity. The order of these activities is defined in a clinical pathway. Moreover, the persons who are supposed to perform the checklist are also predefined in the clinical pathway by assigning each task a potential owner’s role. In each checklist, a checkable item can be defined either as a static text string or as the result of a clinical rule. For example, an item like “Blood samples for cross-typing has been taken” should be applied to every patient without change. Therefore, it is implemented as a static string. However, some patients may have their specific concern which should be reflected in the checklist. For example, the surgeon should be aware of the patient’s renal insufficiency. This item is implemented as a clinical rule like “*IF* Renal Insufficiency is true *THEN* add an item ‘Renal insufficiency noticed”’. As a result, these items only present in the checklist for those who have renal insufficiency.

From the aforementioned checklist implementations, a dynamic checklist should have two main features. Firstly, a dynamic checklist should be process-oriented. SURPASS Digital enables its user overview the status of the whole checking process. Tracebook even goes further. It disseminates checklists to the right users automatically according to predefined clinical pathways. These process-oriented features help clinical users perform the check at the most proper time. Secondly, a dynamic checklist should be patient context-aware. In these cases, each checklist is customized in a patient-specific fashion, so that care-givers are able to focus on each patient. Additionally, patient data and supporting materials are provided to the users together with the checklist. In such a way, users can identify the problems that a dynamic checklist item points out.

These features were broken down to the following modeling requirements. Requirement R1 The dynamic checklist model should support clinical workflow which can be in sequential, parallel or conditional orders and interoperable with other systems. Requirement R2 The dynamic checklist model should support the domain specific type of activity, which has safety guards facing the potential clinical problem. Requirement R3 The dynamic checklist model should support both simple situational-action rule (SAR) and nested rules, i.e., one rule can be used as the action of another rule. Requirement R4 The dynamic checklist model should support expressing the content of safety checklists in such a way that they are associated with relevant clinical activities and specific to every patient when necessary. Requirement R5 The dynamic checklist model should support providing auxiliary information improving users’ efficiency and therefore facilitate the smooth adoption of safety checklists.

#### Solution Domain Investigation: Support in Existing Modeling Approaches and Tools

During the last decades, various languages are developed for modeling health care related processes, esp. guidelines. Among these languages, they can be divided into two categories by their design purpose and application domain [19]. One category is business process modeling language, which focuses on describing activities, roles, resources and their relationships in complex business processes. The other category is guideline modeling language which focuses on decomposition of guideline tasks and clinical logic inside.

For clinical process related modeling approaches, both domain specific modeling languages and extensions to general purpose languages have been studied. Burwitz et al. [20] proposed a domain specific modeling language for modeling clinical pathway.

The approaches of using general purpose modeling languages are focused on business process modeling and notation (BPMN) [21]. BPMN is a standard that offers the most expressive and understandable language at the time of writing this manuscript. The standard also prescribes an interchange format that enables the combination of modeling software and runtime execution software from different industry vendors [21]. The BPMN language was designed to be comprehensible by both IT specialists and professionals. Various other authors treat BPMN as a cost-efficient, rational, standardized, intuitive and flexible instrument for modeling healthcare processes [22]. Besides being expressive and understandable, BPMN is also easy to be extended. The BPMN can be extended in two ways [21]. Firstly, the standard BPMN elements can be extended with additional attributes that can be supported in specific modeling tools and executed by a customized execution engine. Additionally, non-standard elements or artifacts for domain specific purpose can be added to the standard BPMN as extensions. Concrete BPMN implementations already show the feasibility of extending the standard BPMN. For example, jBPM^1^ developers have extended a standard BPMN element, user task, with on-entry and on-exit actions with the purpose of representing actions before and after the execution of a user task [23].

Scheuerlein et al. [22] showed the feasibility of using BPMN to model clinical pathways. Müller et al. [24] developed an extension to BPMN so that shared tasks, which is very common in healthcare processes, can be expressed in the extended BPMN. Hashemian et al. [14] showed the theoretical feasibility of mapping clinical pathway to BPMN. Braun et al. [25] proposed a BPMN extension named BPMN4CP dedicated to representing the clinical pathway in BPMN.

Representation of clinical algorithms is supported in various guideline modeling languages [26]. These languages are all designed with the purpose of representing clinical algorithms as groups of decision logic. Among these languages, Arden, SAGE (standards-based Shareable Active Guideline Environment) and GLIF (GuideLine Interchange Format) share similar ontologies and therefore have comparable semantics [27]. Especially, GLIF has been designed with the purpose of being shareable between organizations [13]. Particularly, de Clercq et al. [28] have developed the Gaston framework, which proposes a domain ontology plus a problem-solving ontology using GLIF’s primitives. A pharmacy decision support system has been developed and widely used together with its pharmacy rules across the Netherlands [29]. The mapping between GLIF and other clinical guideline languages is also possible and well studied [30].

In recent years, industry rule languages have been increasingly applied to clinical domain. For example, Drools was reported in literature [31, 32]. These languages benefit greatly from the open source communities and are developing rapidly.

While studying these modeling languages, model editors as well as execution engines have been developed accordingly. These tools are developed for non-computer experts and enable domain experts to formalize and encode their thoughts directly. To make the encoded knowledge executable for clinical applications, execution engines are also developed. We have practiced with these modeling approaches to build a CABG dynamic checklist set in our prior work [11, 33]. Considering our goal of making the reusable and shareable checklists, reusing standards or widely-accepted models are preferred.

#### Define the meta-model

**Fig. 1:**
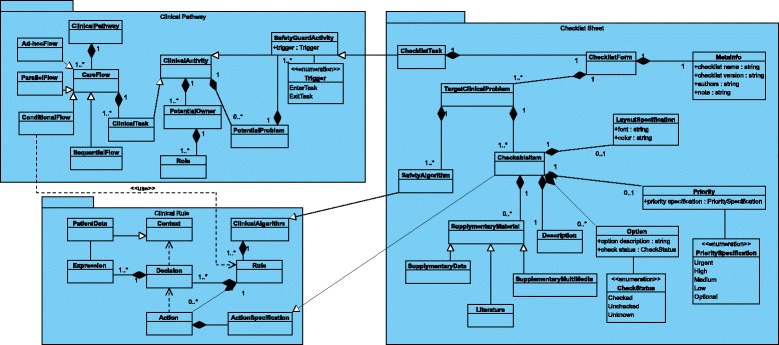
Main classes in the Tracebook meta-model

After having the modeling requirements in mind and learning from aforementioned modeling languages, we categorized dynamic checklist related concepts into three groups, which are process-related concepts, context-related concepts, and concepts describing the format and content of checklists. A meta-model (see Fig. 1) was then defined based on these analyses.

Clinical activity is at the center of all the process-related concepts. Each clinical related task or group of tasks operated in a specific scenario is considered as a clinical activity. Each clinical activity has one or several potential owner, who is responsible for performing this activity. A potential owner has a role indicating his/her specialization. Clinical task is a kind of atomic clinical activity, meaning that this activity can not be decomposed into more detailed activities. These clinical tasks are organized in a clinical pathway, which is a predefined specification of which tasks will be executed in which order. Specifically, the care flow is used to define such orders. These orders include sequential order, parallel order, conditional order and ad hoc order. Each of these types is described by a specific flow pattern, i.e, sequential flow, parallel flow, conditional flow and ad-hoc flow. Clinical activities are always carried out in high pressure and heavy workload situations where errors or potential problems tend to happen. In order to prevent these potential problems, safety guards are needed to protect the activity. A safety guard activity is triggered by a trigger event which indicates something happens while performing the clinical activity. For example, an activity may be supposed to be completed, but in actual practice, it might not proceed due to some reason. In such a condition, the safety guard is triggered, and a safety guard activity will be performed to guarantee the safety of the patient. A safety guard activity is still a clinical activity. So, additional safety guard is still applicable to a safety guard activity if the safety guard activity can not be executed. However, different from another kind of clinical activities, the safety guard activity has a specification to the strategy to be used to protect the safety. The strategy is defined as the algorithm which we describe in the next paragraph.

A clinical algorithm is a specification of how to perform a task. An algorithm has one or more contexts describing its initial input situations. For example, patient data is a kind of context describing the status of a patient. In contrast to context, an algorithm has one or more actions indicating what to do if the given context if fulfilled. The detail of the expected action is described in an action specification. This is a general structure of a clinical algorithm. However, the link between the context and the action is not specified. This link can be a heuristic algorithm, a Bayesian Network model or a clinical rule. In a clinical rule, expressions are used to describe the logic between conditions and actions.

The checklist is used as a reminder to critical steps in a clinical activity. The action of performing a checklist (checklist task) is a safety guard activity which has been defined in the process-related concepts. A checklist form is used as the container of the content of checklist task. Meta-info including title, author, version, etc. is then used to modify the maintenance information of the checklist form. Each checklist form is designed for several target clinical problems. A target clinical problem can be solved by some safety algorithms in the format of a group of checkable items. In each checkable item, there must be a description of the specification of the task. Options are normally needed as indicators of whether the task has been completed or not. However, in some cases, e.g., emergency situations, a checklist is used as a guidance where there is no time nor necessity of marking out the options. Priority is an optional attribute of a checkable item. A checkable item can be ranked up or marked with highlight according to the layout specification if it has a high priority. Lastly, supplementary materials can be applied when needed. The supplementary material can be either supplementary data facilitating that users understand the current patient situation or literature facilitating that users find useful information from the medical literature.

### Case study

After having developed the meta-model, we validated its feasibility by two case studies. The meta-model was mapped to specific modeling languages according to the requirements of hospitals.

#### Case selection

In order to validate the meta-model, two checklists were implemented in our study. One is a coronary artery bypass graft (CABG) peri-operative checklist in a Dutch hospital, Catharina Hospital. The other is a percutaneous coronary intervention (PCI) peri-operative checklist in a Chinese hospital, People’s Liberation Army (PLA) General Hospital.

Both of these two checklists are designed for peri-operative phase, which is a comprehensive process needing collaboration among various clinical roles. Additionally, in the peri-operative process, a variety of clinical rules are applied to check if proper treatments are given. These features made these two checklists suitable as our case study objects.

These two hospitals have different IT infrastructures and preferences. In Catharina Hospital, the rule-based clinical decision support system, Gaston, has been used over a decade. Both medical workers and IT staff in the hospital had a strong will to reuse the system and clinical rules in it. Thus, Gaston was implemented to edit and execute clinical rules. However, Gaston is not available in the Chinese hospital. Therefore, we choose Drools, which is powerful and widely used open source rule engine. Considering the availability of tool support and the requirement of communicating with medical staff, we chose BPMN to represent the clinical process for both of these cases.

#### Map the meta-model to mature modeling languages

We mapped the process-related dynamic checklist concepts with the concepts in BPMN modeling language. The basic ideas of the clinical process can be well-supported in BPMN with the exact BPMN concepts without extension. A clinical pathway which relates to several checklists can be considered to be equivalent to a process in BPMN. It is a container of the description of the control flow. A care flow is such a control flow. Specifically, each category of care flow has its own map in BPMN. The sequential flow means two tasks have to be executed one by one. This can be described by using a sequence flow in BPMN, which is an arrow that connects two tasks. The parallel flow can be represented by adding a parallel gateway in BPMN. The parallel gateway can represent that several tasks (and their succeeding tasks) are executed simultaneously. The conditional flow enables selecting different tasks according to the specific condition. This can be represented with exclusive gateway and inclusive gateway in BPMN based on the converging criteria. Ad-hoc flow represents a batch of tasks which do not have a predefined execution order. This feature is supported by ad-hoc subprocess in BPMN.

To represent specific safety checklist concepts, some items in BPMN need be extended. We derived the clinical user task from the user task in BPMN. However, different from the user task, safety guard activities are required in order to prevent potential problems that affect patient safety. A safety guard activity (e.g. using a checklist) is activated by a trigger. For example, at the time when a clinical user task is completed, a checklist should be given to some clinical practitioners to make sure the task has been done properly. These concepts are not supported in the BPMN standard. Fortunately, these requirements are partly supported in BPMN based modeling tools and execution engine, e.g. jBPM and BizAgi. BizAgi provides a feature enabling executing extra tasks at the time entering and exiting a task. Specific to a checklist, the activity of performing a safety checklist is a kind of clinical user task, that is protected by one or more safety guard(s). In this way, a checklist is integrated into the clinical process represented in BPMN. The mapping relationships are summarized in Table 1.

**Table 1:** Mapping dynamic checklist concepts to BPMN concepts

Checklist concept	BPMN concept	Explanation
Clinical pathway	Process	A clinical pathway is a kind of process.
Care flow	Sequence flow, Gateways, Ad-hoc subprocess	A care flow specifies how two or more clinical tasks are connected.
Sequential flow	Sequence flow	Two tasks are executed sequentially.
Parallel flow	Parallel gateway	Two tasks are executed simultaneously.
Conditional flow	Exclusive gateway, Inclusive gateway	The succeeding task can only be executed if some conditions are met.
Ad-hoc flow	Ad-hoc subprocess	Tasks are executed without a specific order.
Clinical activity	Task	A clinical activity is a clinical-oriented task.
Clinical task	User task	A clinical task is a clinical-oriented user task.
Potential owner	Potential owner	A potential owner is an expected owner of a clinical task.
Role	Resource role	A role is the clinical role of a potential owner. A potential owner may have one or more roles.
Safety guard activity	User task	Safety guard activity is a clinical task dedicated to preventing potential medical errors.
Potential problem	N/A	Potential problem describes what possibly might go wrong while performing a clinical task.
Trigger	N/A	A trigger is a description of when should a safety guard activity be executed.

Then, we mapped the rule-related concepts to the concepts in Gaston and Drools. A clinical algorithm is usually adopted from clinical guidelines describing the best practice. In Gaston, the concept is presented as clinical guideline. Whereas in Drools, which is a general purposed rule language, the concept can be mapped to a rule file. An algorithm can be further broken down to several rules. Gaston’s guideline concept has a nested mechanism that a guideline can be decomposed to several sub-guidelines and a sub-guideline is also a guideline. In Drools, each rule file contains several rules. The algorithm describes the decision logic in response to some specific patient conditions. The decision logic is usually represented in the flow chart form in Gaston, including decision step and action step. These two Gaston concepts map to decision and action, respectively. Similarly, Drools has conditional element and action element to map with these concepts. Patient context is the patient data item used for specifying under which condition a decision should be made. Both data item in Gaston and fact in Drools can be mapped to this concept. An action specification specifies what to do in an action. Gaston has a dedicated element action specification for this. In Drools, a property name value in action can be used. The full mapping relationships are summarized in Table 2.

**Table 2:** Mapping dynamic checklist concepts to Gaston and Drools concepts

Checklist concept	Gaston concept	Drools concept	Explanation
Clinical algorithm	Nested guideline	Rule file	A clinical algorithm is usually adopted from clinical guidelines describing the best practice.
Rule	Guideline	Rule	A clinical algorithm is usually adopted from clinical guidelines describing the best practice.
Context	Data item	Fact	Patient context is the patient data item used for specifying under which condition a decision should be made.
Decision	Decision step	Conditional element	Decision is a step in an algorithm describing which branch to choose under a certain context.
Action	Action Step	Action element	Action is a step describing the inference result once a decision is made.
Expression	Gaston expression	Conditional element Eval.expression	Expression is used for expressing the criteria for a decision.
Action specification	Action specification	Action Element.value	An action specification specifies what to do in an action step.

#### Develop support systems

**Fig. 2:**
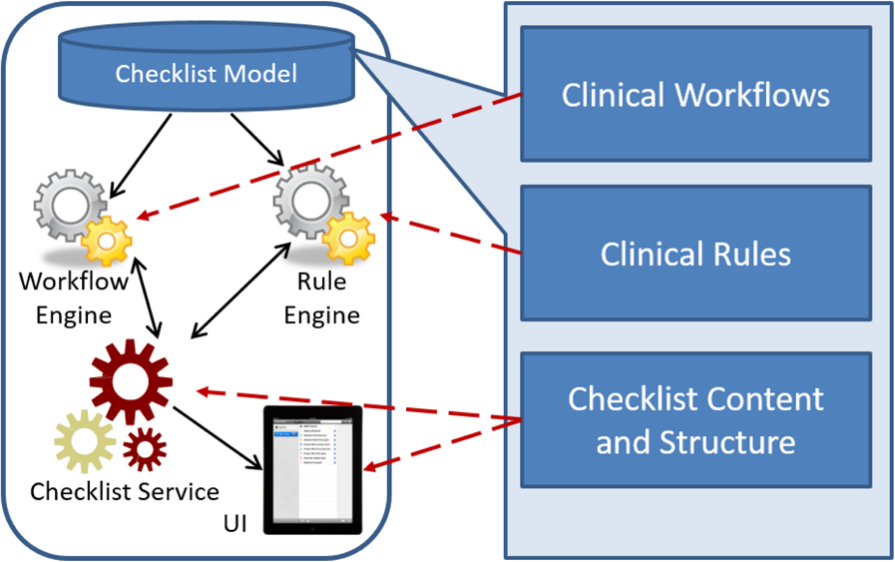
System architecture of the prototype system based on the meta-model

To validate our methodology, we developed a prototype dynamic checklist decision support system, Tracebook, for executing dynamic checklist models [11]. In accordance to the meta-model, Tracebook has three main components for model execution and a UI component for the interaction with users (see Fig. 2). The workflow engine is designed to support the clinical pathway. In this part, we interfaced with BizAgi Express business management system’s APIs^2^. The rule engine is used to support clinical rules. In this part, we interfaced with Gaston and Drools, respectively. We implemented the checklist engine to deal with the interoperability between the workflow engine and rule engine based on our checklist model. The checklist engine also sends checklists to the UI renderer in XML format. The UI renderer uses XSLT to interpret the XML into HTML 5 format and show them to users as checklists.

This system has been implemented in both Catharina Hospital and PLA General Hospital, interfacing with the hospital EMR systems in both hospitals. Considering different IT infrastructures, these two implementations chose Gaston rule engine and Drools rule engine, respectively. As requested by clinical users, the user interface was adapted and fine-tuned in each hospital accordingly.

## Results

Following the aforementioned approach, we implemented two dynamic checklists in two hospitals. In this section, we illustrate the implementation results.

### The CABG peri-operative checklist in the Dutch hospital

Interviews with care-givers were carried out to document the peri-operative care process and related rules. We took a two-step approach to model the peri-operative checklist from the documentation. The first step is to model the care process, which is described in the care pathway package in the meta-model. Then, checklist sheets and related rules were modeled, which is described in the checklist sheet package and clinical rule package, respectively.

**Fig. 3:**
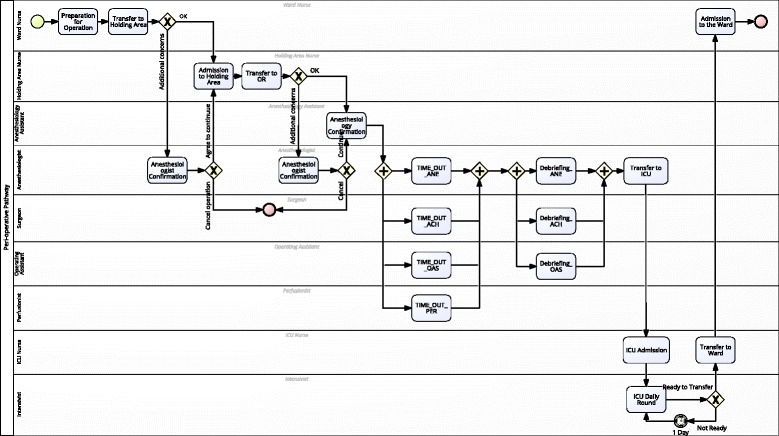
A CABG peri-operative pathway model

In the first step, we modeled the peri-operative care workflow in BPMN language with BizAgi Process Modeler (see Fig. 3). Nineteen clinical activities were identified in the care process. They were modeled as user tasks in the BPMN language. Nine roles are responsible for these tasks. Every task is assigned to a specific role. Swim lane is used for specifying the roles that should be assigned to each checklist sheet. List of persons who belong to this role (i.e., potential owners) is edited and managed by Tracebook. The list also includes the contact information of these persons. By doing these, the workflow engine can distribute a checklist to the related persons and further give them reminders. Parallel gateway is used for the split and join of parallel tasks, and the exclusive gateway is used for branching and synchronizing conditional flows. However, the condition of branching should be considered here, but the detail of logic is a part of task layer. Each checklist is triggered when a clinical user enters a clinical task.

**Fig. 4:**
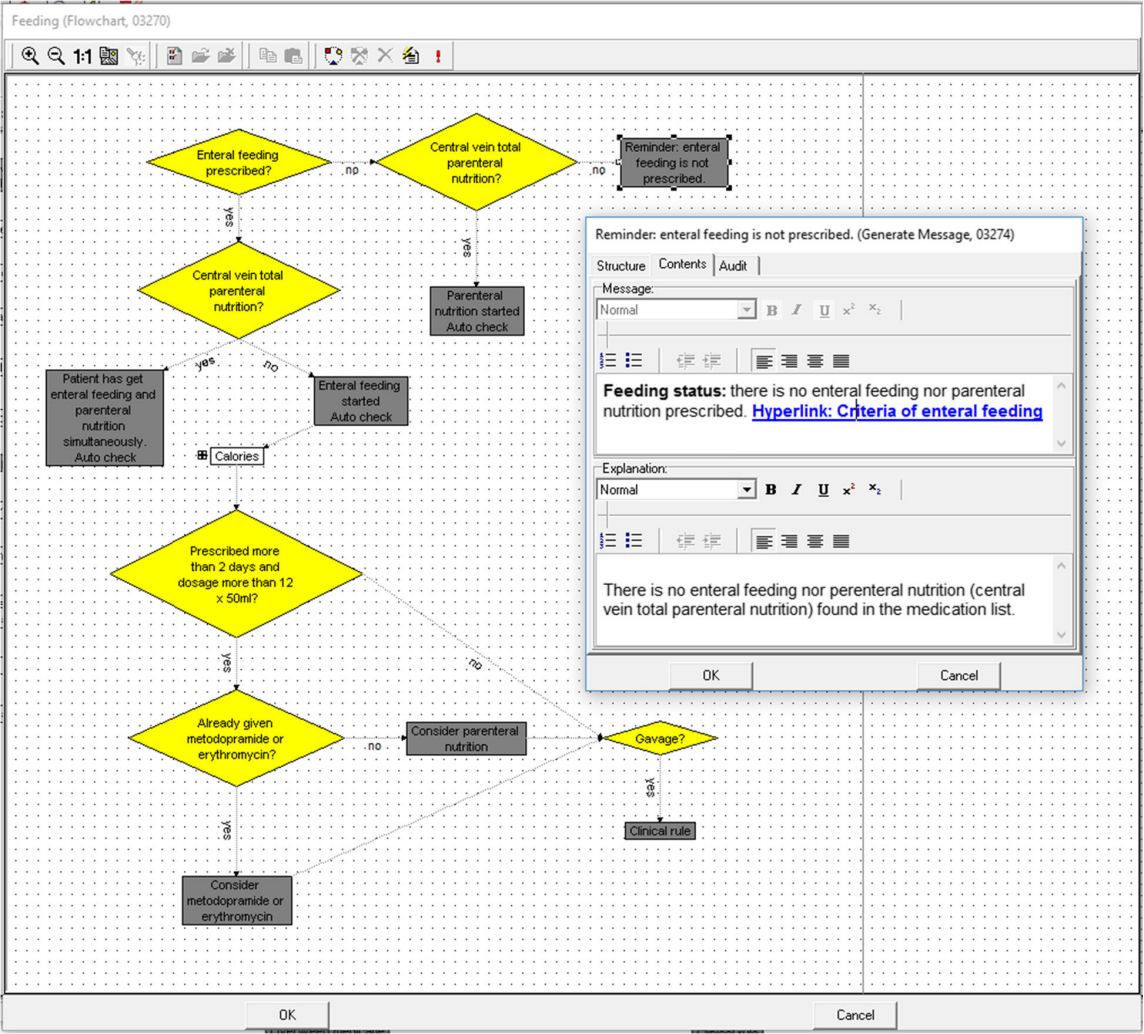
An item related to feeding in the ICU daily round checklist

In the second step, checklist sheets and related rules were modeled. We used Gaston editor as the knowledge acquisition tool for these two steps. A checklist form contains several target clinical problems, which are modeled as sub-guidelines in Gaston. Each checkable item is related to an action in Gaston. In each task, we define the content of the checkable item (including the link to patient data) and explanation of the item if needed. Additionally, decision logic is used to highlight items, provide personalized items and pre-check items for double-checking. The user interface and an example of the rule are included in Fig. 4.

For the system development, we interfaced with BizAgi Express business management system’s APIs and Gaston. We implemented the checklist engine to deal with the interoperability between the workflow engine and rule engine based on our checklist model. The checklist engine also sends checklists to the UI renderer in XML format. The UI renderer uses XSLT to interpret the XML into HTML 5 format and show them to users as checklists.

### The PCI checklist in the Chinese hospital

**Fig. 5:**
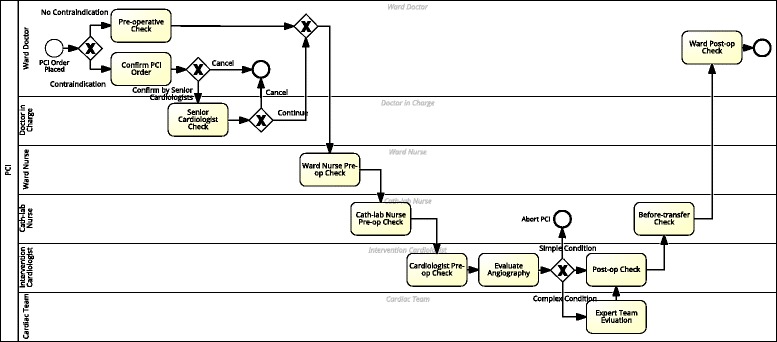
The PCI peri-operative care pathway

For the PCI checklist in the Chinese hospital, we took a similar approach. We implemented the process model also in the BPMN language (Fig. 5).

**Fig. 6:**
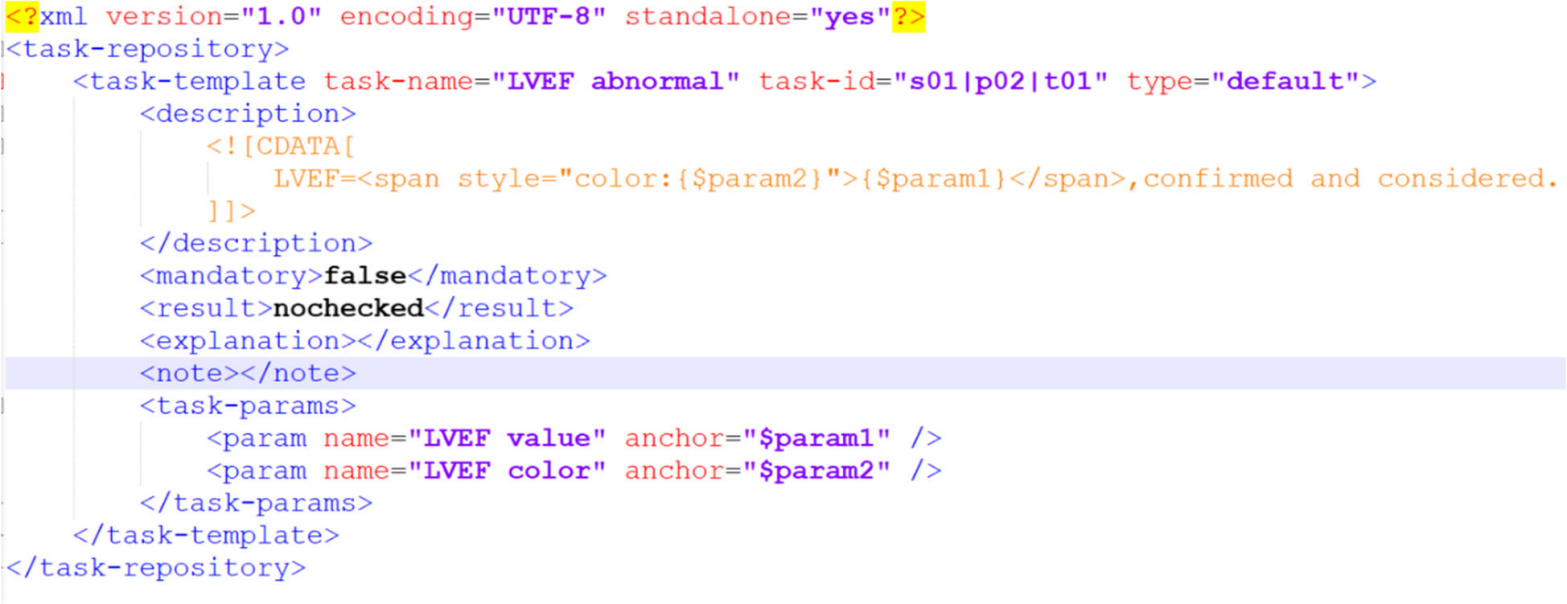
Example of a predefined checklist item

**Fig. 7:**
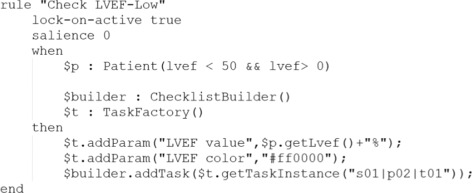
Example of a clinical rule related to a checklist item

Different from the first case, we used another approach to implement clinical rules and the content of checklists. In Drools, rules are presented in the form of “WHEN *condition* THEN *action*”. These rules are edited in a plain-text editor. Checkable items are produced and modified in the action clauses in these rules. We take the left ventricular ejection fraction (LVEF) as an example. Based on the meta-model, the item is defined in the form illustrated in Fig. 6. Then, this item is associated with a clinical rule defined as Fig. 7. When the patient’s LVEF is less than 50, the rule will be fired, and a patient specific item is alerting the doctor will be generated. Specifically, the abnormal LVEF value is marked with the red color so that the doctors can find it at their first glance.

**Fig. 8:**
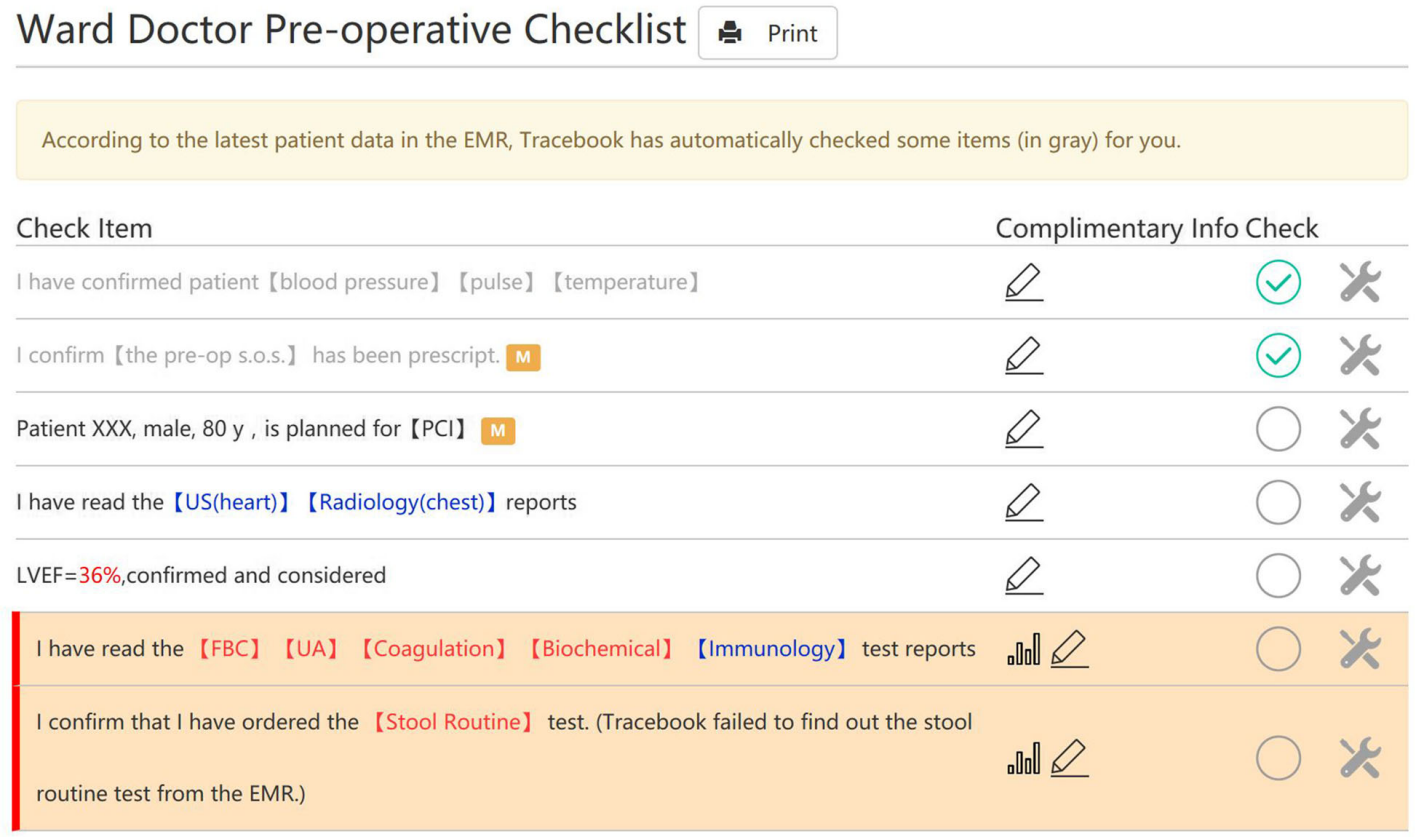
Screen shot of a fragment of checklist used in the Chinese hospital

This model is also executed by our Tracebook system in the Chinese hospital. A fragment of the pre-operative checklist in the system can be found in Fig. 8. The check items in gray are automatically checked by the system, according to predefined clinical rules. The items end with a mark “M” are items of high priority. Data items in the checklist items are linked to patient data in the EMR. Items in red color indicate abnormalities are found in these items. In the given example, the patient’s LVEF is 36, which is less than 50. Therefore, an item alerting the situation is provided.

## Discussion

### Experiences gained in the two implementations

While implementing these two cases, there are several advantages while using our meta-model. The model driven approach significantly reduced the workload of developing checklist support systems. In both the Dutch hospital and the Chinese hospital, the content of checklists has been updated continuously without changing the source code of the Tracebook system. This shortens the iteration loop of developing dynamic checklists in hospitals. The meta-model enables its developers to choose mature modeling language, tools, and execution engines. By doing so, clinical workers, informaticists and hospital IT staff all can take advantage from it. Indeed, in our case study, our clinical participants can fully understand care pathways in BPMN and clinical algorithms in Gaston. IT infrastructure in hospitals can be reused to speed up the development and implementation.

Both Gaston and Drools are used in our implementations. While modeling clinical rules for dynamic checklists, these two languages, together with their related tools, have their advantages and disadvantages. Gaston represents clinical rules in a flowchart fashion, which is easy for clinical experts to understand. Clinical rules in Drools are organized as groups of SARs, which is easy for maintain by informaticists. However, both of these two languages can fully capture the algorithms provided by clinical experts. When it comes to the tool support, Gaston has a user-friendly rule editing tool, which makes trained clinicians able to edit rules by themselves. Such nice tools are yet lacking for Drools. Therefore, informaticists have to write the rule by themselves and confirm them with clinical experts.

### Shareability and reusability enabled by the meta-model

Both our cases use BPMN as the language for expressing clinical processes. Previous research shows the feasibility of mapping BPMN to other executable languages such as BPEL and Petri-net [34, 35]. Thus, our model should be able to implement other languages. Our ongoing work is to implement Case Management Model and Notation (CMMN) to make other case studies.

Though this work is an extension to BPMN and GLIF, with our proposed conceptual model, it is also possible to apply the methodology to other business modeling languages (even descriptive languages, e.g., CMMN [36]) which have the concept of task. Moreover, the conceptual model also serves as a guideline of using the built-in rule module and UI designer in commercially available business modeling tools, e.g., BizAgi, jBPM, and Activiti.

### The necessity of using more than one modeling language

In our case studies, we use more than one modeling language to implement dynamic checklists. In the Chinese hospital case, it is clear that it is impractical to use Drools to represent the care processes. However, in the Dutch hospital case, both BPMN and GLIF are used. Both of them are wide-spread modeling languages in industry and healthcare. These two languages share lots of similarities in their syntax and modeling constructs. Moreover, both of these two languages have been used to represent clinical processes. In addition to process, rules are supported in both languages as well. However, both processes and rules are supported at different levels by these two languages.

Comparing with BPMN, GLIF does not support the user, role, event, etc. The ability to represent workflow concepts of GLIF and BPMN have been studied thoroughly by Peleg et al. As a conclusion, though it is possible to model clinical processes with GLIF, extra constructs are needed or have to be added in the runtime [37].

Regarding rules, BPMN has defined a limited interface and left the implementation to the vendors. Therefore, the content of rules is not defined in the BPMN model [38]. This is exactly the strength of GLIF, in which rich constructs and expressions can be used to represent rules, especially clinical rules.

From the above-mentioned analysis, it is clear that by solely using BPMN or GLIF, the whole idea of the checklist can be supported only to some extent, but not completely. To explicate every concept that is needed for a checklist, these two languages should be combined and integrated.

### The strengths and short-comings of extending existing modeling languages

Both BPMN and GLIF have good visualization supported by various applications. Drools is a popular rule language in the industry in recent years. The first strength of these languages is the widely-available modeling tools and execution engines. Existing models in their support systems which are already running in the hospital can be easily adapted and reused. Secondly, domain users are already familiar with these languages so that the adoption cost is lower than newly-developed languages. It might be argued that there is a shortcoming in that our approach is difficult due to the complexity of learning and using two languages instead of a single unified language. Note. However, that usually clinical processes and specific checklists are different concerns by different roles in a hospital. The management board is usually the main stakeholder of standardizing the clinical process so that the care processes carried out in their hospital is standard and controllable. Regarding a checklist, it is the concern of a specific department where the checklist related activities are carried out. As a result, two groups of people are working on one group of checklist set. These two groups of people can work with their familiar language and integrate their work together as a whole at the very end. Only limited and affordable marginal efforts need to be taken.

### Potential advantages of the meta-model

One potential advantage of this work is that it gives a platform independent model which can be applied to multiple modeling languages and execution engines by model transformation [39]. Many workflow modeling languages and clinical guideline languages have been practices in healthcare over decades. People would expect to benefit from these mature approaches and tools. Additionally, hospitals always have their own legacy clinical decision systems which often contains rule engines. In this case, they want to have an easy way to reuse them for making their checklists. Since our meta-model considers both the general requirements of dynamic checklists and the features of existing modeling approaches, it can be mapped to several modeling languages by defining proper mapping rules. Therefore, these mature languages can be reused for modeling dynamic checklists.

Another potential advantage is that international standards are used for representing checklist knowledge. That is to say, knowledge sharing between different facilities would be easy if they also follow standards in their information systems, which is a trend in system implementation in hospitals.

### Limitations

In clinical settings, it is possible that some people work together as a group for some certain scenario, but they have their duty in parallel. In the proposed meta-model, we consider these group of people as a composed role which includes all the actors involved. However, this concept is not well delivered in the execution phase. This is because workflow execution engines have only one actor for every task. One way to solve this problem is to duplicate those tasks to different actors. However, this is against the idea that people should physically work together for those tasks.

The layout of checklist influences the acceptance of checklist implementation a lot. Different tasks may have different priorities and marked with different colors. However, layout problem in our meta-model is yet not considered. This is because that we hold the idea that we should split the content of knowledge and the representation to the end users. In this way, it is more flexible to be applied and distributed to different organizations.

## Conclusion

The checklist is a widely used technique to help improve medical quality and reducing avoidable errors. Clinical research shows the great power of checklist. However, due to the static nature of paper-based checklist and simple digital checklist, the adherence to checklist still has room for improving. One way to improve the adherence is to make checklist more dynamic, integrating seamlessly into the clinical workflow and making it more context aware. Checklist modeling is the first step of making such a dynamic checklist.

In this paper, we proposed a framework of reusing existing modeling languages and tools to model dynamic checklists. We analyzed the modeling requirements for the checklist and proposed a three-layer framework of checklist modeling. For each layer, based on the requirements, we analyzed and selected concepts from existing formal languages and reuse these concepts and their relationships in our model. We also implemented BPMN and Gaston to validate our meta-model by modeling and implementing the CABG peri-operative checklist into a dynamic checklist which can integrate into the hospital EMR system.

## Abbreviations

AMC: Academic Medical Center
API: Application program interface
BPMN: Business process modeling and notation
CABG: Coronary artery bypass graft
CDSS: Clinical decision support system
CMMN: Case management model and notation
CPMS: Clinical pathway management system
CPOE: Computerized provider order entry
EMR: Electronic medical record system
GLIF: GuideLine interchange format
HTML: HyperText markup language
ICU: Intensive care unit
LVEF: Left ventricular ejection fraction
OR: Operating room
PCI: Percutaneous coronary intervention
PLA: Chinese People’s Liberation Army
SAGE: Standards-based shareable active guideline environment
SAR: Situation-action rule
SURPASS: Surgical patient safety system
XML: Extensible markup language
XSLT: Extensible stylesheet language transformations

## Footnotes

1 See http://www.jbpm.org/.

2 See http://wiki.bizagi.com/.
